# Insane Asylum—the Alleged Abuses in Their Administration

**Published:** 1881-03

**Authors:** 


### INSANE ASYLUMS—THE ALLEGED ABUSES IN THEIR ADMINISTRATION.

The management of asylums for the insane has become a subject of severe criticism, within a few years, in professional as well as political circles, and public sentiment has been rendered keenly sensitive upon this and kindred matters. While this discussion as to the administration of other asylums of the State has convinced candid minds that there was much ado about nothing, the spark, thus fanned by the current of popular feeling, has been kindled into quite a flame by certain statements arising from an irresponsible source, in regard to the treatment of patients in the State Asylum, located in this city.

We refer to this subject at this time, because we believe the profession is unanimous in the opinion that the treatment of this important class of diseases in institutions specially devoted to this purpose, is an absolute necessity. As a profession, therefore, we have a direct interest, not only in the establishment of Insane Asylums, but also in the scientific and humane treatment of the unfortunate patients, who may be confined therein by reason of the mental infirmities with which they chance to be afflicted. We believe also, that the State has secured, for the administration of these important interests, men of the highest professional ability and attainments. The Asylum, located in this city, has been placed under the able superintendency of Dr Judson B. Andrews, for many years connected with the Utica Insane Asylum, whose reputation in this department of medicine extends far beyond the confines of our own State, and for whose honesty and uprightness of aim and purpose we are able to bear the most emphatic testimony. In common with the profession, the public are also interested not only in the establishment of these agencies, but also in their wise administration. The State recognizes the fact that its safety can be secured only by the fullest protection it affords to the individuals of which it is composed; hence, the erection of these costly edifices, and the annual expenditure of large sums for their maintenance. But such agencies, though controlled by the highest administrative wisdom, are necessary liable to the imperfections and uncertainties incident to all human instrumentalities. The visible participation of angelic agencies has not thus far been vouchsafed either to the State or to individuals in any of the manifold concerns of this world.

The statements above referred to in regard to the administration of the Buffalo Insane Asylum were unfortunately made the subject of public comment through the secular press. Briefly, they consist of charges of inhuman and cruel treatment by two of the subordinate officials towards certain patients, confined in the Asylum. These statements were given to the public solely on the authority of a subordinate, named Churchhill, without having first formally presented them to the Superintendent.

Now, we do not bring a judicial mind to the examination of these charges, nor to the task of analyzing the testimony by which it is attempted to support them, but we venture the opinion, after carefully perusing Churchill's (the accuser's) sworn statement before the Commissioner of Lunacy, that in his effort to make out a case he endeavors to prove too much, and by so doing, without any corroborative evidence to substantiate his accusations, impairs, if he does not wholly vitiate, the credibility of the entire charge.

The Asylum authorities can be actuated by but one motive in dealing with this matter. To them, neither the accuser nor the accused, have any special significance in comparison to the greater interest involved in the proper care of the insane, a trust for which they are directly responsible. We have the fullest confidence that merited rebuke and prompt dismissal will at all times be visited upon every assistant in the institution for any dereliction of duty, or abuse of authority; and to this end we commend the prompt manner in which the accusations have been met by the President of the Board of Trustees and the Superintendent in at once seeking the fullest investigation, and by inviting the most thorough inspection of the institution and its manner of treating the insane. In this, the public will be satisfied that the authorities aim to do their full duty in the responsible work committed to their care, and to correct whatever abuses may exist.

Our city, in common with the profession and the public at large, have a great interest and a just pride in the success of this institution. We desire to assist the Superintendent in prosecuting the work on which he has entered with so much zeal and wise judgment. We feel that such attacks are subversive of good disciples and may lead to serious embarrassments in securing competent and reliable assistants. But, on the other hand we are assured that the disposition already manifested of meeting whatever issues may be presented in a manly way, will inspire the confidence of all in an institution, which is destined, at an early day, to be the foremost of its kind in the land.

